# Histological Estimates of Ovariole Number in Honey Bee Queens, *Apis mellifera*, Reveal Lack of Correlation with other Queen Quality Measures

**DOI:** 10.1673/031.011.8201

**Published:** 2011-07-02

**Authors:** Jeffrey T. Jackson, David R. Tarpy, Susan E. Fahrbach

**Affiliations:** ^1^ Department of Biology, Wake Forest University, Winston-Salem, NC 27109, USA; ^2^ Department of Entomology, Campus Box 7613, North Carolina State University, Raleigh, NC 27695-7613, USA

**Keywords:** nurse cell, oocyte, ovary, trophocyte

## Abstract

Published estimates of the number of ovarioles found in the ovaries of honey bee, *Apis mellifera* L. (Hymenoptera: Apidae) queens range from 100 to 180 per ovary. Within the context of a large-scale study designed to assay the overall quality of queens obtained from various commercial sources, a simple histology-based method for accurate determination of ovariole number was developed and then applied to a sample of 75 queens. Although all 10 commercial sources evaluated provided queens with ovariole numbers within the expected range, ovariole number was found to vary significantly across sources. Overall, and within most of the individual samples, there was no correlation of ovariole number with other morphological attributes such as thoracic width, wing length, or wet weight. Queens from two of the sources, however, displayed a significant negative relationship between wet weight and ovariole number. This study provides baseline data on ovariole number in commercial honey bee queens in the United States at a time when honey bee populations are declining; the method described can be used in studies relating ovariole number in queens to egg production and behavior.

## Introduction

Ovaries in hymenopteran females are divided into elongated tubular ovarioles (Martins and Serrão 2004). Many families (Andrenidae, Megachilidae, Halictidae, Colletidae, and Melittidae) have three ovarioles per ovary, while the Apidae have four or more ovarioles. In the case of European honey bee queens, *Apis mellifera* L. (Hymenoptera: Apidae), however, there are upwards of one hundred ovarioles per ovary, whereas workers of this species typically have fewer than 10 ovarioles per ovary (Snodgrass 1956; Velthuis 1970; Chaud-Netto and Bueno 1979). This striking difference in ovariole number (and corresponding reproductive capacity) between queens and workers is a result of programmed cell death of ovarian tissue during worker development of larvae not fed a diet of royal jelly (Reginato and Cruz-Landim 2002; Reginato and Cruz-Landim 2003).

*Apis mellifera* workers of different genotypes can differ in their number of ovarioles (Thuller et al. 1998). Variation in worker ovariole number is correlated with several behavioral traits, including the sugar concentration of nectar loads and the display of the retinue response to queen mandibular pheromone (Linksvayer et al. 2009; Kocher et al. 2010). Worker ovariole number is therefore a phenotype that can provide insight into the evolution of the female castes in social insects (Amdam et al. 2006; Amdam et al. 2009). Workers with more ovarioles per ovary are also more likely than workers with fewer ovarioles to become the dominant egg layers in queenless colonies (Makert et al. 2006).

**Figure 1.**
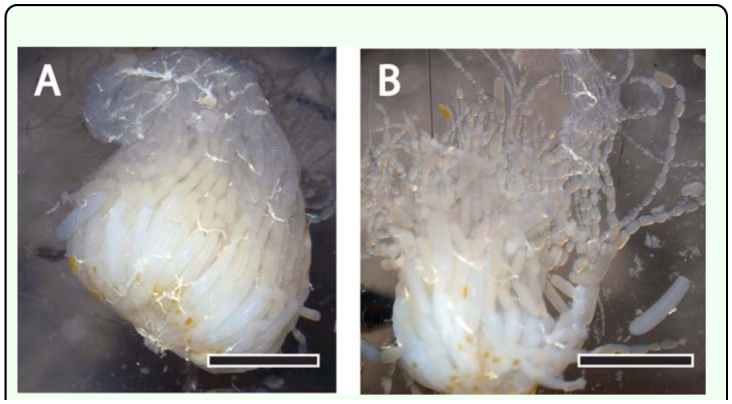
Manual dissection of an ovary of the *Apis mellifera* queen. (A) Intact ovary removed from the queen's abdomen via a dorsal midline incision. Scale bar, 500 µm. (B) Beginning of the destructive process of counting ovarioles in a freshly dissected queen ovary. Scale bar, 500 µm. High quality figures are available online

These and other studies of worker ovariole number depend upon their accurate quantification. This is typically accomplished by spreading the individual ovarioles in a drop of saline; the observer then views the freshly dissected ovary through a stereomicroscope and counts the ovarioles directly. Making an accurate determination of the number of ovarioles in a queen ovary, however, is more challenging because the typical heavily tracheated queen ovary contains 10 or more times the number of ovarioles as a typical worker ovary (Figure 1). Moreover, the reported range for ovariole number per ovary in honey bee queens is wide, from 100 to 180 ovarioles per ovary (Snodgrass 1956).

Because of the difficulty in counting numerous ovarioles, it is unclear what proportion of this reported variation reflects genetic or environmentally induced developmental differences and what proportion simply reflects the difficulty of obtaining accurate counts from freshly dissected tissue.

The functional significance of this variation among queens is also unclear. Do queens with more ovarioles lay more eggs than queens with fewer ovarioles? It has been argued that the very large number of ovarioles found in a typical queen bee renders the exact number meaningless: (Grosch et al. 1977). In contrast to this view, studies of insects with a smaller number of ovarioles, such as *Drosophila melanogaster*, have documented a positive relationship between ovariole number and egg production (David 1970; Bouletreau-Merle 1978), although studies using measures of fitness other than egg production have not found a relationship between ovariole number and fecundity (Wayne et al. 1997). These and other unresolved issues regarding the importance of ovariole number in honey bee queens are not merely of theoretical interest, but are also relevant to the beekeeping operations on which many food crops depend. There has long been an awareness of the existence in honey bee queens of ovarian anomalies that reduce egg laying, although the causes in most cases were unknown (Fyg 1964; Camazine et al. 1998). A recent survey of U.S. beekeeping operations reported that beekeepers ranked “poor queens” as leading the top suspected causes for colony losses (van Engelsdorp et al. 2008). Moreover, exposure to the acaricide coumaphos has been shown to reduce ovary weight in honey bee queens (Haarmann et al. 2002).

Examination of freshly dissected tissue results in a destructive count, as the ovary is inevitably destroyed in the process of counting individual ovarioles. Counts based on light microscopic examination of cross sections prepared from fixed, embedded, and sectioned ovaries require more labor to produce, but preserve a permanent record of ovarian structure. This paper describes a wax section-based method for estimating ovariole number in honey bee queens using simple equipment found in most histology laboratories. This method complements previously described methods based on embedding in methacrylate resin or araldite for the preparation, respectively, of thin and ultrathin sections of ovarioles (Dedej et al. 1998, Tanaka and Hartfelder 2004), and will therefore be useful when less detail is required and when the goal is primarily to count ovarioles. Because the procedure was developed to assess variation in ovariole number in a large sample of naturally mated North American honey bee queens purchased anonymously from commercial sources as part of a systematic study of many parameters of queen reproductive potential, other measures of queen quality were recorded prior to dissection (Delaney et al. 2011).

## Materials and Methods

### Bees

Naturally open-mated queen honey bees from various commercial queen producers were ordered in the winter of 2007 for arrival during the spring of 2008. Queens were shipped directly to an anonymous address at the Lake Wheeler Honey Bee Research Facility at North Carolina State University. Ten commercial queen sources were selected semi-randomly to sample different regions of the United States (particularly the Southeast and Western regions, where queen producers are clustered). Twelve queens were purchased from each, and all queens were of the common ‘Italian’ stock. Upon arrival, two queens from each source were introduced into established colonies following standard methods and allowed to lay so that adult offspring could be collected for paternity analysis (see Delaney et al. 2011). The remaining ten queens from each source were banked without an opportunity to lay (Laidlaw and Page 1997) in strong colonies until further processing. Non-experimental queens were acquired from Brushy Mountain Bee Farms (Moravian Falls, NC) to provide freshly dissected ovaries for photography.

### Dissection, fixation, embedding

All queens were weighed to the nearest 0.1 mg after immobilization by freezing for ∼4 min at -20° C. While immobilized, head and thorax widths were measured to the nearest 0.1 mm using digital calipers. Each forewing was removed and taped to a data sheet for later measurement of wing lengths. Queens were euthanized by decapitation, pinned onto a dissection plate, and covered with RNAlater® (Applied Biosystems, www.appliedbiosystems.com) so that tissues could be collected for subsequent analyses. The abdomen of each queen was then dissected while being viewed through a stereomicroscope (zoom magnification adjusted as needed). After removal of the spermatheca, the right and left ovaries were carefully removed with forceps and transferred immediately to alcoholic Bouin's fixative prepared by combining 150 ml 80% ethanol, 60 ml commercially prepared formaldehyde, 15 ml glacial acetic acid, and 1 g of saturated picric acid solution (Presnell and Schreibman 1997) for fixation by immersion. Although some sources recommend the addition of 1% DMSO to this fixative, this was not found to be necessary to obtain excellent fixation of honey bee tissues. Trials with ovaries from queens not included in this study demonstrated that, unless required for other studies, the RNAlater® was not required to obtain good tissue preservation. The right ovary was defined as the ovary on the right side of the queen pinned ventral side down and anterior forward on the dissecting dish. After overnight fixation at room temperature, dehydration was completed by transferring the ovaries to 100% ethanol (two changes, 15 minutes each). Dehydrated ovaries were immersed into a 1:1 solution of 100% ethanol:xylene for one hour, then transferred to xylene for storage until infiltration with molten Paraplast (Leica, www.leica-microsystems.com) in a 60° C oven (two changes of molten Paraplast, approximately 60 minutes each in duration). The quality of the infiltration was improved by cooling the freshly infiltrated tissue to room temperature and then re-melting the Paraplast prior to embedding in 7 × 7 × 5 mm disposable base molds (Shandon, Thermo Fisher, www.thermoscientific.com). The ovaries are easy to manipulate during processing because they are colored bright yellow by the picric acid in the Bouin's fixative. To produce a consistent plane of section, ovaries were carefully oriented in the base molds using heated forceps and the process was viewed through a stereomicroscope. The ovary was placed in the corner of the mold for support and the lateral oviduct was placed touching the base of the mold, so that it would be the first structure sectioned. The tissue was supported in the proper orientation by forceps grasping the distal tip of the ovary as the Paraplast cooled. It is always possible to return a poorly-oriented embedded specimen to the 60° C oven for re-melting and re-embedding. Right and left ovaries from individual queens were tracked throughout the procedure to check for evidence of asymmetry in ovariole number, as has been previously reported for *Apis mellifera* worker ovaries (Chaud-Netto and Bueno 1979).

### Sectioning and staining

The wax blocks containing the embedded ovaries were sectioned using a rotary microtome with heavy duty high profile disposable microtome blades (C.L. Sturkey, Inc., www.sturkey.com). Short ribbons of 10 µm-thick sections were mounted in a pool of water on the non-frosted portion of Superfrost Plus slides (Fisher, www.fishersci.com). The slide was then transferred to a slidewarmer set at 48° C. When the warmed sections had visibly flattened, the water was drained from the slide. The slide was then cooled briefly on the counter (for approximately 1 minute), blotted firmly with bibulous paper, and then returned to the slidewarmer overnight. Slides were stored at room temperature in a covered box until staining. The first sections collected were from the broadest part of the ovary and contained oocytes with large amounts of yolk and large trophocytes. Later sections contained oocytes with less yolk and smaller trophocytes and were overall easier to count, so this level was generally selected for mounting and staining. Far distal sections were not collected and counted as this might cause short ovarioles to be missed. Immediately prior to staining, paraffin was removed from sections by immersion in xylene (three changes, 5 minutes each) and re-hydrated in a graded series of ethanols of descending concentrations, 5 minutes per change (100%, 100%, 95%, 70% with lithium carbonate, 50%, and 30%). The lithium carbonate was included to remove the yellow picric acid from the tissue (Presnell and Schreibman 1997). After a brief dip in distilled water, sections were then stained for 2 minutes in trichrome stain prepared by adding phosphotungstic acid (1 g), orange G (2 g), aniline blue WS (1 g), and acid fuchsin (3 g) to 200 ml distilled water (Cason 1950; Humason 1972) and differentiated in 95% ethanol prior to dehydrating in 100% ethanol, clearing in CitriSolv (Fisher Scientific, www.fishersci.com), and coverslipping with Permount (Fisher Scientific). Slides were stored flat for a week at room temperature prior to microscopy to ensure adequate drying of the Permount mounting medium.

### Microscopy, image acquisition, and ovariole counting

Images of three non-consecutive cross-sections were acquired with a Q-Imaging Retiga 4000R digital camera attached to a Leica MZ16 FA motorized stereomicroscope system using reflected light illumination. Using this stereomicroscope with Leica 10x eyepieces (Leica, www.leica-microsystems.com) and 0.63x Planapo objective (Leica) enabled an entire ovary cross-section to be captured in a single field of view. Images were previewed, acquired, processed, and analyzed using Image Pro Plus 6.2 (Media Cybernetic, www.mediacy.com). Images (2048 × 2048 pixels) were saved as .tif files. Ovariole counts made using Image Pro Plus were facilitated by using the computer mouse to place user-defined tags manually over each counted ovariole, so that no ovariole was double-counted. This procedure yielded a permanent record of each ovary and facilitated re-counting of ovarioles by another observer. Slides were coded so that counts were made blind to source of the queen.

### Sample sizes and statistical analysis

**Figure 2.**
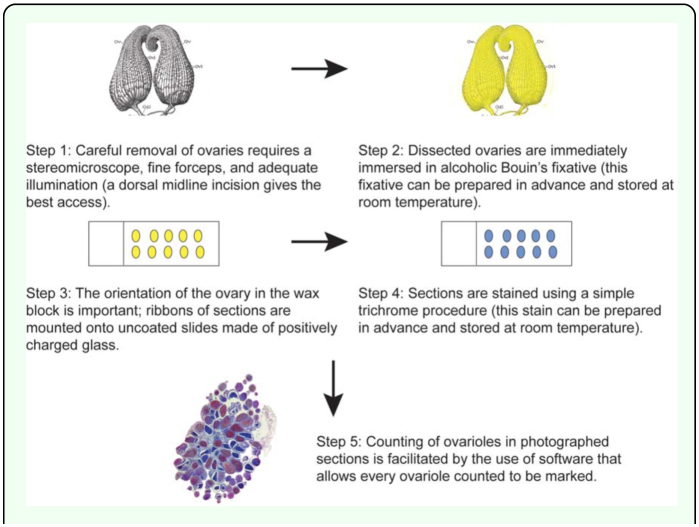
Schematic diagram summarizing the flow of work. Diagram of *Apis mellifera* ovaries modified from Snodgrass (1956). High quality figures are available online

The 10 sources that supplied queens used in this study were blindly coded by the letters A through J to ensure anonymity of the commercial sources. The number of samples from each source that provided both right and left ovaries for histology and subsequent analysis were 8 (Source A), 9 (Source B), 6 (Source C), 10 (Source D), 3 (Source E), 9 (Source F), 10 (Source G), 10 (Source H), 4 (Source I), and 6 (Source J) for a total of 75 right and left pairs. Queens were omitted from the study if one or both of their ovaries was damaged during dissection or subsequent tissue processing; in this study 25% of the ovaries (25/100) did not produce useable sections. The primary reason for loss of data was damage during the initial dissection. Data were analyzed using GraphPad Prism 5.01 for Windows (www.graphpad.com). Because three nonconsecutive sections were counted for each ovary, analyses could be based on either the middle number (median) of the three sections counted per ovary or the maximum number of ovarioles counted per ovary. The latter was typically preferred based on the reasoning that presence of an ovariole in one section was sufficient evidence of the ovariole's existence. This number is referred to as “maximum count” in the reporting of the data. Figure 2 summarizes the flow of work. Only data that passed tests for normality were subjected to parametric analysis: in all other cases, non-parametric tests were applied.

## Results

### Identification of ovarioles in cross-sections

**Figure 3.**
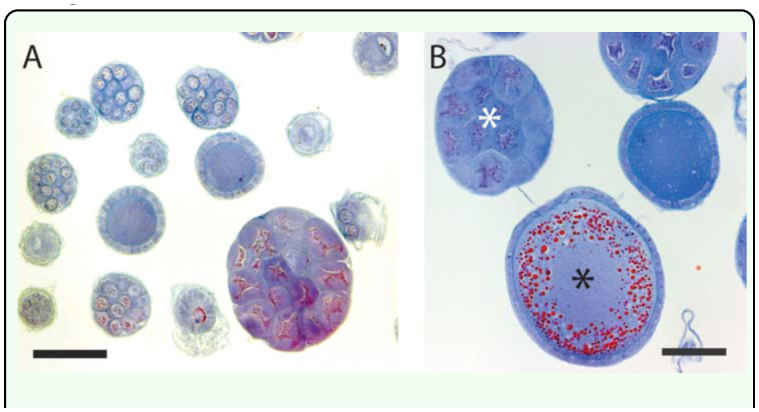
Examples of *Apis mellifera* ovarioles of naturally mated queens viewed in cross section. (A) Small diameter ovarioles are sampled when sections are collected at roughly the midpoint of the ovary. Scale bar, 100 µm. (B) Larger diameter ovarioles, including oocytes filled with abundant yolk, are visible if sections are collected proximal to the lateral oviduct. Black asterisk indicates a cluster of trophocytes; white asterisk indicates a cross section though an oocyte. Scale bar, 100 µm. High quality figures are available online

*A. mellifera* ovarioles are meroistic (sister cells, called nurse cells or trophocytes, remain connected to the oocyte as it moves down the ovariole to the lateral oviduct) and polytrophic (trophocytes are contained within the follicle rather than remaining in the germarium). Because of this, ovarioles have an unambiguous appearance in cross-section: either the oocyte itself or a cluster of trophocytes is observed ensheathed by a layer of follicle cells (Figure 3). Inexperienced observers may occasionally count tracheae or Malpighian tubules inadvertently captured in the dissection as ovarioles, but accurate, reproducible counts can be achieved with less than an hour of practice (tracheae have a distinctive ridged appearance; Malpighian tubules have a smaller diameter than ovarioles). Misidentifications can also be minimized by careful dissection and by counting ovarioles in sections distal to the lateral oviduct (approximately midway between the lateral oviduct and the thread-like terminal filament), where the contents of the follicles are not as large and the individual ovarioles are less crowded together.

### Ovariole counts

**Figure 4.**
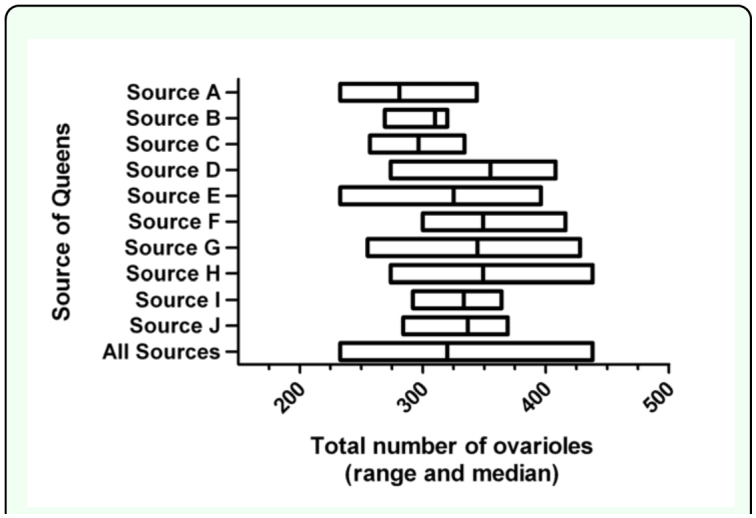
Range and median total number of ovarioles (sum of counts of right and left ovaries) in a sample of 75 *Apis mellifera* queens obtained from commercial sources in the United States in Summer 2008. The combined data for the entire sample is also shown. High quality figures are available online

The median number of ovarioles per queen (based on the sum of the right and left maximum counts for each individual queen) across all sources was 320. The range was broad, extending from a low of 233 to a high of 438. The results of a Kruskal-Wallis nonparametric one-way ANOVA revealed that the medians varied significantly by source (Kruskal-Wallis statistic 22.64, number of groups = 10, *p* < 0.01; Figure 4). The effect was weak, however, as a Dunn's Multiple Comparison Test for *post hoc* pairwise comparisons did not reveal any significant differences in rank sum. Because all tests for normality (Kolmogorov-Smirnov test, Shapiro-Wilk test, and the D'Agostino and Pearson omnibus test) indicated that the data were normally distributed (a > 0.05), it was judged appropriate to compare mean number of ovarioles using a one-way ANOVA and the Newman-Keuls Multiple Comparison Test. The one-way ANOVA revealed that the means varied significantly by source (F_10,df2_ = 2.80, *p* < 0.01) and that the mean for Source A was significantly lower than the means for Sources D, G, and H (p < 0.05). These results were nearly identical if, instead of summing the maximum counts, the middle value (median) of the three counts per ovary for each individual queen was summed (Kruskal- Wallis statistic 20.70, number of groups = 10, p = 0.0141).

**Figure 5.**
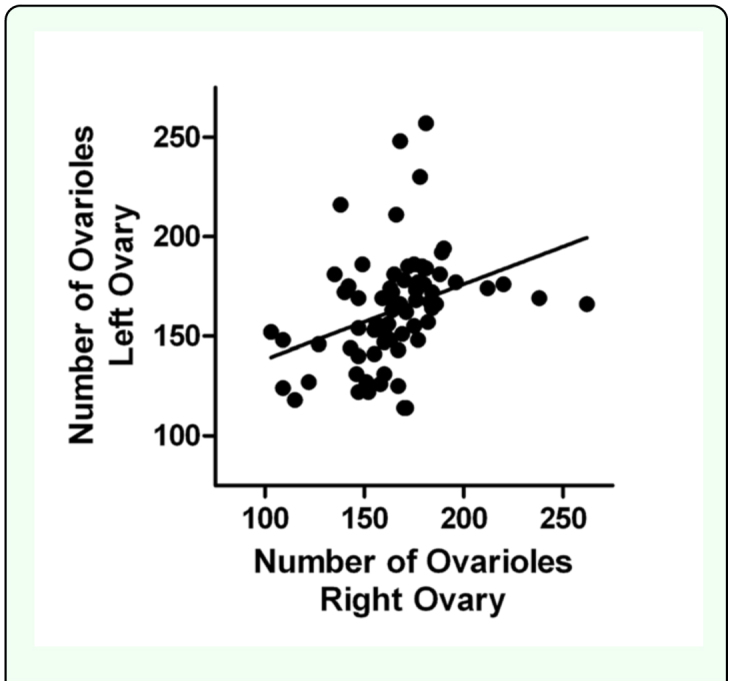
Number of ovarioles in the right and left ovaries of a sample of 75 *Apis mellifera* queens obtained from commercial sources in the United States in 2008. Independent of source, the right ovary typically contained more ovarioles than the left ovary. High quality figures are available online

Because the right and left ovaries were tracked through the entire procedure, it was possible to assess the potential asymmetry in ovariole number within each queen. Linear regression analysis revealed a slight asymmetry that favored the right ovary (r^2^ = 0.12, F_df1,df2_ = 9.96, *p* < 0.005; Figure 5), but the difference was independent of queen source (Kruskal-Wallis statistic = 5.43, number of groups = 10, p = 0.8).

### Correlations of ovariole counts with other possible measures of queen quality

The relationship of ovariole number to several measures of body size, sperm count, and viral load was determined as described in Delaney et al. (2011). No correlation was found with thoracic width, head capsule width, and length of either wing, either across all sources or within sources (data not shown). As expected, thoracic width was positively correlated with wing length (Spearman r = 0.2986, *p* = 0.0134). Linear regression analysis revealed that the length of the right wing was highly correlated with the length of the left wing (r^2^ = 0.8048, F = 263.9, *p* < 0.0001). There was also no correlation across the entire sample between wet weight of the queens and total number of ovarioles (Spearman r = -0.226, *p* = 0.053) or between sperm count and number of ovarioles (Spearman r = 0.091, *p* = 0.451). There was no correlation between the presence of Deformed Wing Virus (DWV) and number of ovarioles (Spearman r = 0.005, *p* = 0.966). With the exception of two sources, there was no correlation between ovariole number and wet weight within source. In both Source D and Source G, however, there was a significant negative relationship between ovariole number and wet weight (Source D: Spearman r = -0.794, *p* < 0.005; Source G: Spearman r = -0.709, *p* < 0.05).

## Discussion

Concerns over threats to *A. mellifera* populations are global and include questions about trends in the reproductive potential of commercial queens. The present study concentrated on one possible aspect of queen quality: ovariole number. Ovariole counts were performed on ovarian cross sections obtained from a large sample of queens obtained from commercial breeding operations in the United States. The data confirm the existence of substantial variation in ovariole number in *Apis mellifera* queens. The data also revealed that, although the total number of ovarioles differed slightly by source, all queens sampled had ovariole counts within the expected range. There was a tendency for the right ovary to contain more ovarioles than the left ovary, and there was no correlation of ovariole number with any other measure of queen reproductive potential. In particular, it should be noted that the overall size of the queen, whether gauged by wet weight or measures of body size such as thoracic width, was not a good predictor of ovariole number (see Eckert, 1934; Hatch et al. 1999). In fact, in the two sources in which wet weight and ovariole number were significantly related, higher weights were associated with lower ovariole numbers. Because the queens were banked (not laying), these differences may reflect differential feeding of the banked queens rather than a relationship between ovariole number and wet weight.

Because there are few historical records of *A. mellifera* anatomy that could serve as a basis for comparison, it is difficult to assess whether contemporary queens have more or fewer ovarioles than past generations of queens. Furgala (1962) found that 7.1% of 465 queens in packaged bees had “underdeveloped ovaries”, and Camazine et al. (1998) found that 12% of 325 commercial queens had fewer than five developed eggs per ovary. Eckert (1934) found an average of 318.8±1.21 ovarioles per queen, but his samples were raised experimentally and not commercially produced. Of the current queen population, we found that 7.5% of the queens had fewer than 125 ovarioles per ovary (250 total per queen). While we hesitate to make direct comparisons with the above studies, the present study provides a valuable snapshot of the current status of honey bee queen ovariole number in North America as being comparatively and sufficiently high.

The simple and efficient method described here can be readily applied to other populations and is well-suited to preserve archival records of ovariole counts. Ovariole counts provide insight into honey bee queens complementary to direct measures of reproductive activity performed on adult queens (e.g. stored sperm counts, number of drone fathers among their offspring) because any non-genetic variation in ovariole number reflects impacts of pathogens, chemical contaminants, and other factors at the late larval stage when final ovariole number is determined. Parallel studies using direct measures of reproductive activity on queens revealed that, although commercially sourced queens in the United States are overall sufficiently inseminated and mated with an appropriate number of drones, there is significant variation in physical traits and mating quality (Camazine et al. 1998; Delaney et al. 2011).

The finding of a difference between the number of ovarioles in the right and left ovary is in accord with a previously reported right-left difference in the number of ovarioles in the ovaries of *A. mellifera* workers (Chaud-Netto and Bueno 1979) and queens (Eckert 1934), the former reported that the number of ovarioles was typically greater in the left ovary. In the latter study and our sample of *A. mellifera* queens, the right ovary almost always contained more ovarioles. Whether this right/left discrepancy is a methodological or a biological difference is impossible to determine given the brevity of the earlier publications. It should be noted that this is a covert asymmetry that is not reflected in any apparent asymmetry in external morphology, as indicated by a striking correlation of right and left wing length. The functional significance of this asymmetry, if any, is unknown.

Many unanswered questions regarding ovariole number in *A. mellifera* queens can now be confidently answered using the simple method described in this paper. These include experimental studies of genetic and environmental factors determining ovariole number, assessment of a possible relationship between ovariole number and egg production, and the relationship between ovariole number in queens and their worker offspring. The finding of a negative relationship between ovariole number and wet weight in two of the samples suggests that queens with more ovarioles may direct more resources to their offspring than queens with fewer ovarioles, and should be examined directly under conditions in which queens are able to lay and age and access to nutritional resources are controlled. Given recent discoveries of links between ovariole number and worker behavior, it is also relevant to ask if differences in queen behavior might also reflect differences in ovariole number.

## References

[bibr01] Amdam GV, Csondes A, Fondrk MK, Page RE (2006). Complex social behavior derived from maternal reproductive traits.. *Nature*.

[bibr02] Amdam GV, Ihle KE, Page RE, Arnold AP, Etgen AM, Fahrbach SE, Rubin RT, Pfaff DW (2009). Regulation of honey bee (*Apis mellifera*) life histories by vitellogenin.. *Hormones, Brain and Behavior*.

[bibr03] Bouletreau-Merle J (1978). Ovarian activity and reproductive potential in a natural population of *Drosophila melanogaster*.. *Oecologia*.

[bibr04] Camazine S, Çakmak I, Cramp K, Finley J, Fisher J, Frazier M, Rozo A (1998). How healthy are commercially-produced U.S. honey bee queens?. *American Bee Journal*.

[bibr05] Cason JE (1950). A rapid one-step Mallory-Heidenhain stain for connective tissue.. *Stain Technology*.

[bibr06] Chaud-Netto J, Bueno OC (1979). Number of ovarioles in workers of *Apis mellifera adansonii* and *Apis mellifera ligustica*: a comparative study.. *Journal of Apicultural Research*.

[bibr07] David JR (1970). Le nombre d'ovarioles chez *Drosophila melanogaster*: relation avec fécondité et valeur adaptative.. *Archives de Zoologie Experimentale et Generale*..

[bibr08] Dedej S, Hartfelder K, Aumeier P, Rosenkranz P, Engels W (1998). Caste determination is a sequential process: effect of larval age at grafting on ovariole number, hind leg size, and cephalic volatiles in the honey bee (*Apis mellifera carnica*).. *Journal of Apicultural Research*.

[bibr09] Delaney DA, Keller JJ, Caren JR, Tarpy DR (2011). The physical, insemination, and reproductive quality of honey bee queens (*Apis mellifera* L.).. *Apidologie*..

[bibr10] Eckert JE (1934). Studies in the number of ovarioles in queen honeybees in relation to body size.. *Journal of Economic Entomology*.

[bibr11] Furgala B (1962). Factors affecting queen loss in package bees.. *Gleanings in Bee Culture*.

[bibr12] Fyg W (1964). Anomalies and diseases of the queen honey bee.. *Annual Review of Entomology*.

[bibr13] Grosch DS, Kratsas RG, Petters RM (1977). Variation in *Habrobracon juglandis* ovariole number. I. Ovariole number increase induced by extended cold shock of fourth-instar larvae.. *Journal of Embryology and Experimental Morphology*.

[bibr14] Haarmann T, Spivak M, Weaver D, Weaver B, Glenn T (2002). Effects of fluvalinate and coumaphos on queen honey bees (Hymenoptera: Apidae) in two commercial queen rearing operations.. *Journal of Economic Entomology*.

[bibr15] Hatch S, Tarpy DR, Fletcher DJC (1999). Worker regulation of emergency queen rearing in honey bee colonies and the resultant variation in queen quality.. *Insectes Sociaux*.

[bibr16] Humason GL (1972). *Animal Tissue Techniques*.

[bibr17] Kocher SD, Ayroles JF, Stone EA, Grozinger CM (2010). Individual variation in pheromone response correlates with reproductive traits and brain gene expression in worker honey bees.. *PLoS ONE*.

[bibr18] Laidlaw HH, Page RE (1997). *Queen Rearing and Bee Breeding*..

[bibr19] Linksvayer TA, Rueppell O, Siegel A, Kaftanoglu O, Page RE, Amdam GV (2009). The genetic basis of transgressive ovary size in honeybee workers.. *Genetics*.

[bibr20] Makert GR, Paxton RJ, Hartfelder K (2006). Ovariole number — a predictor of differential reproductive success among worker subfamilies in queenless honeybee (*Apis mellifera* L.) colonies.. *Behavior, Ecology and Sociobiology*.

[bibr21] Martins GF, Serrão JE (2004). A comparative study of the ovaries in some Brazilian bees (Hymenoptera; Apoidea).. *Papéis Avulsos de Zoologia São Paulo*.

[bibr22] Presnell JK, Schreibman MP (1997). *Humason's Animal Tissue Techniques*.

[bibr23] Reginato RD, Cruz-Landim C (2002). Morphological characterization of cell death during the ovary differentiation in worker honey bee.. *Cell Biology International*.

[bibr24] Reginato RD, Cruz-Landim C (2003). Ovarian growth during larval development of queen and worker of *Apis mellifera* (Hymenoptera: Apidae).. *Brazilian Journal of Biology*.

[bibr25] Snodgrass RE (1956). *Anatomy of the Honey Bee*..

[bibr26] Tanaka ED, Hartfelder K (2004). The initial stages of oogenesis and their relation to differential fertility in the honey bee (*Apis mellifera*) castes.. *Arthropod Structure and Development*.

[bibr27] Thuller RHC, Malaspina O, Bueno OC, Chaud-Netto J (1998). Number of ovarioles in workers descendant from crossings between Africanized and Italian honeybees, *Apis mellifera* L.: Comparison among backcrosses and ancestors colonies.. *Anais da Sociedade Entomológica do Brasil*.

[bibr28] vanEngelsdorp D, Hayes J, Underwood RM, Pettis J (2008). A survey of honey bee colony losses in the U.S., Fall 2007 to Spring 2008.. *PLoS ONE*.

[bibr29] Velthuis HHW (1970). Ovarian development in *Apis mellifera* worker bees.. *Entomologia Experimentalis et Applicata*.

[bibr30] Wayne ML, Hackett JB, Mackay TFC (1997). Quantitative genetics of ovariole number in *Drosophila melanogaster*. I. Segregating variation and fitness.. *Evolution*.

